# A Review of Centrifugal Testing of Gasoline Contamination and Remediation

**DOI:** 10.3390/ijerph8083496

**Published:** 2011-08-24

**Authors:** Jay N. Meegoda, Liming Hu

**Affiliations:** 1 Department of Civil and Environmental Engineering, New Jersey Institute of Technology, University Heights, Newark, NJ 07102, USA; 2 State Key Laboratory of HydroScience and Engineering, Department of Hydraulic Engineering, Tsinghua University, 1 East Zhongguancun Road, Beijing 100084, China; E-Mail: gehu@tsinghua.edu.cn

**Keywords:** leaking underground storage tanks, gasoline contamination, centrifugal modeling, soil vapor extraction, *in-situ* air sparging, remediation and reuse

## Abstract

Leaking underground storage tanks (USTs) containing gasoline represent a significant public health hazard. Virtually undetectable to the UST owner, gasoline leaks can contaminate groundwater supplies. In order to develop remediation plans one must know the extent of gasoline contamination. Centrifugal simulations showed that in silty and sandy soils gasoline moved due to the physical process of advection and was retained as a pool of free products above the water table. However, in clayey soils there was a limited leak with lateral spreading and without pooling of free products above the water table. Amount leaked depends on both the type of soil underneath the USTs and the amount of corrosion. The soil vapor extraction (SVE) technology seems to be an effective method to remove contaminants from above the water table in contaminated sites. *In-situ* air sparging (IAS) is a groundwater remediation technology for contamination below the water table, which involves the injection of air under pressure into a well installed into the saturated zone. However, current state of the art is not adequate to develop a design guide for site implementation. New information is being currently generated by both centrifugal tests as well as theoretical models to develop a design guide for IAS. The petroleum contaminated soils excavated from leaking UST sites can be used for construction of highway pavements, specifically as sub-base material or blended and used as hot or cold mix asphalt concrete. Cost analysis shows that 5% petroleum contaminated soils is included in hot or cold mix asphalt concrete can save US$5.00 production cost per ton of asphalt produced.

## 1. Introduction

The worldwide boom in automobile sales following World War II was closely followed by the construction of thousands of gasoline stations. At these new stations, steel underground storage tanks (USTs) were installed to store the fuel. The average life expectancy of a steel tank was 30 to 50 years, depending on the corrosion of the steel. Since the early 1980s, corrosion of steel tanks, along with faulty installation and operation has resulted in an increasing number of incidents of groundwater contamination by gasoline. Leaking gasoline tanks now represent a significant public health hazard. Leaking gasoline tanks can also present the risk of fire and explosion because vapors from leaking tanks can travel through sewer lines into buildings.

Leaking USTs are called LUSTs. Because most LUST sites are contaminated by gasoline, benzene, toluene, ethylbenzene, and xylenes (together referred to as the BTEX compounds) constituents of gasoline are the typical contaminants of concern of LUST sites.

- Benzene is the most hazardous of these compounds. Its EPA Maximum Contaminant Level (MCL) is 5 parts per billion (ppb). Long-term exposures to benzene in drinking water at levels above the MCL increase the risk of cancer.
- Toluene and ethylbenzene are not considered carcinogenic (cancer-causing). Their MCLs are 1.0 and 0.7 parts per million (ppm). Over the long term, however, toluene and ethylbenzene damage the liver, kidneys, and central nervous system.
- Xylenes are a mixture of compounds (*ortho*-, *meta*-, and *para*-xylene) with two methyl (−CH_3_) groups attached to a benzene ring. Xylenes also affect the liver, kidneys, and nervous system, but they are not considered nearly as hazardous as the first three—the MCL for total xylenes is 10 ppm.

A slow leak from a typical 10,000 gallon gasoline storage tank at the neighborhood service station would be virtually undetectable to the station operator but still quite hazardous to nearby groundwater supplies. The hazards of gasoline are mainly attributable to the BTEX compounds. The benzene content of typical gasoline is 0.76% by mass (gasoline composition). A spill of 10 gallons of gasoline (only 0.1% of the 10,000 gallon tank, a quantity undetectable by manual gauging and inventory control) contains about 230 grams of benzene. The EPA’s Maximum Contaminant Level (MCL) for benzene is 5 parts per billion (ppb), or 5 micrograms per liter, in drinking water. The density of gasoline is about 0.8 g/mL, so the benzene in a 10 gallon gasoline leak can contaminate about 46 million liters, or 12 million gallons of water!

**Figure 1.**
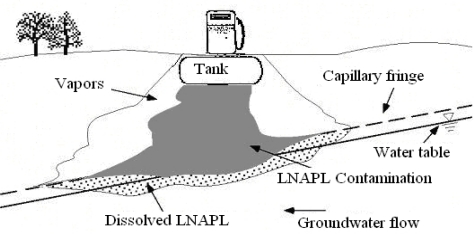
LNAPLs release and subsequent migration [4].

The majority of USTs store petroleum products (gasoline, diesel, heating oil, kerosene, jet fuel, *etc.*), but many other substances classified as hazardous by the Resource Conservation and Recovery Act and the Comprehensive Environmental Response, Compensation, and Liability Act (“Superfund”) are stored in USTs. Gasoline type contaminants are often regarded as Light Non-Aqueous Phase Liquids (LNAPLs). LNAPLs’ transport in subsurface system is a complicated process [1–3], as shown in Figure 1. When LNAPLs are spilled they enter the unsaturated zone under gravity. Upon encountering a capillary fringe, the LNAPL forms a pancake-like layer above the saturated zone. Groundwater flowing past the floating LNAPL dissolves soluble components, forming a dissolved plume down-gradient of the LNAPL zone [4].

The USEPA ACT 40 CFR-280 and the May 1990 amendment to that regulate USTs and enlist the deadlines and financial responsibilities for USTs. The owners of all USTs, including those currently in operation, those taken out of operation after January 1974 but not removed from the ground, or those installed in the future, must register the UST with the appropriate state agency specifying the age, size, type, location and uses of the UST. The above statue also mandates that land owners and anyone who may be involved in site contaminations must develop plans on how to deal with:

- Corrosion and leak protection in tanks.
- Provisions for spills during transportation and operation.
- Monitoring and Testing tanks in service.

In order to prevent releases due to structural failure, corrosion, or spills and overfills for as long as the UST system is used to store regulated substances, all owners and operators of new UST systems must meet the following requirements. Each tank must be properly designed and constructed, and any portion underground that routinely contains product must be protected from corrosion. Hence the tank should be constructed of fiberglass-reinforced plastic or constructed of steel and cathodically protected.

Soil and groundwater contamination due to LUSTs is currently one of the major environmental problems in the world. In order to develop remediation plans one must know the extent of gasoline contamination. Once the extent of gasoline contamination is known, one must know optimal remediation procedure. Hence the objective of this review manuscript is to review the state of the art on centrifugal modeling that describes the extent of gasoline contamination and to provide optimal remediation procedures to mitigate the gasoline contamination.

## 2. Size and Shape of Contamination Plume

Laboratory scale batch or column experiments are not effective in simulating the extent of *in-situ* gasoline contamination. On the other hand, performing pilot field experiments to investigate the effectiveness of remediation technologies is costly and time consuming. The geotechnical centrifuge has proven to be a robust tool to simulate full-scale geo-environmental problems [5–9]. Arulanandan *et al.* [5] developed scaling laws used in simulating geo-environmental problems. Geotechnical centrifuge modeling can be used to represent full-scale prototypes under normal field conditions and simulate the movement and remediation of contaminants. A 1/*N* scale model tested at *N* times centrifugal acceleration of the gravity experiences stress conditions identical to those in the prototype. Centrifugal modeling is an innovative way to simulate the extent of gasoline contamination, where the prototype time is scaled as *N*^2^ based on the scaling laws [5]. Also the use of a centrifuge can be a powerful physical modeling technique to study the long-term pollutant transport in soils [10].

### 2.1. Centrifugal Modeling

One of the earliest uses of a centrifuge in geo-environmental research was proposed by Arulanandan *et al.* [5]. They successfully modeled the pollutant transport process in saturated soils. Nimmo [11] proved that unsaturated flow can be scaled in a centrifuge. Cooke and Mitchell [6] verified that groundwater flow and moisture suction phenomena can be modeled by a centrifuge. Data obtained on the migration of a non-reactive contaminant have indicated that centrifuge modeling may provide a useful means to study contaminant transport in the unsaturated zone. Nakajima *et al.* [12] found that mechanical dispersion can be modeled for low fluid velocity. Illangasekare *et al.* [13] conducted model tests to investigate the 1-D movement of LNAPLs through unsaturated soils following a surface spill under a gravity of 20 *g*. A reasonably sharp front was obtained between the advancing front and the displaced fluids. Mitchell and Stratton [14] modeled a surface spill of light oil in dry medium sand and investigated the extent of the contamination. Hu *et al.* [15] simulated soil venting extraction process for remediation of LNAPLs contaminated soils using centrifuge technique. These studies show that centrifuge modeling technique has the potential to study of the long-term transport of LNAPLs in unsaturated soils.

The migration of LNAPLs in unsaturated soils is a complicated process. The centrifuge model is effective in simulating processes dominated by gravitation. A good technique for checking scale effect is the “modeling of models” [5,16]. This technique is particularly useful when no prototype is available to verify the model test results. With this technique, centrifuge models of different scales are tested at appropriate accelerations such that they can correspond to the same prototype. The models should predict the same behavior and thus provide a useful internal check on the modeling procedure. Some authors have reported similitude in centrifuge modeling tests of LNAPLs migration in unsaturated soils at relatively low gravitational levels. Chang *et al.* [1] directly measured gasoline concentrations after a centrifuge test by collecting soil samples for gas chromatograph analysis. Gravelle *et al.* [17] and Knight and Mitchell [7] described the influence of two different constant rates of release on the penetration of the LNAPLs into an unsaturated granular medium. They modeled the release of 1,000 L of silicon oil into unsaturated fine sand. A reproducible unsaturated moisture-suction profile was established and similar plumes at scale models of 15 *g* and 30 *g* were reported. Esposito *et al.* [18] studied a 2-D spill of LNAPL from a transparent container that enabled direct visual observation of the oil infiltration. These tests took into account the influence of porosity on the migration of the LNAPL into the unsaturated sand and the spreading of the LNAPL at the saturated-unsaturated interface. The test results indicated good agreements of the unsaturated soil profile and the extent of contamination at both 20 *g* and 30 *g*. Culligan *et al.* [19] reported the preferential flow of NAPLs in dry sands. Centrifuge modeling of NAPL transport behavior was reported by Kechavarzi *et al.* [20,21], Hu *et al.* [22], Soga *et al.* [23], Lo *et al.* [24–26] by higher *g*-level tests, and the feasibility of the centrifuge modeling of LNAPL transport was verified by the modeling of models technique. Culligan and Soga [27] gave the general introduction about the application of centrifuge modeling technique in NAPL transport problems.

### 2.2. Simulation of LUST under Stationary Groundwater Table

**Figure 2.**
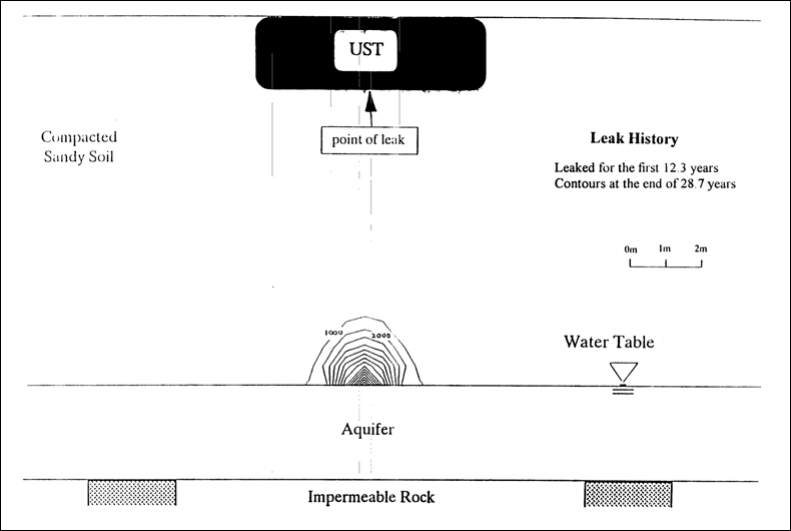
Gasoline contours in ppm after 28.7 years of leaking into a silty soil [1].

Figure 2 shows the contours of total gasoline fraction (concentration) in a silty soil expressed as ppm at the end of 28.7 years after leaking started [1]. The leaked gasoline moved as a front and accumulated as a pool of free product above the capillary fringe. Furthermore, a substantially higher fraction (concentration) of gasoline was observed directly below the leakage source. The gasoline in the mound decreased thickness with increasing radial distance from the center. Also these contours were symmetrical about the source. The gasoline was non-existent at the source of leakage because it was exhausted after 12.3 years of leaking. Thus, the hydrocarbon fraction (concentration) decreased from the top of the aquifer towards the UST. The residual gasoline fractions within the silt layer shown in Figure 2 were below 0.01% or 100 ppm. The above data suggest that the gasoline infiltrated the soil as a front and became accumulated above the capillary fringe. Lo *et al.* [24–26] confirmed the above observations.

**Figure 3.**
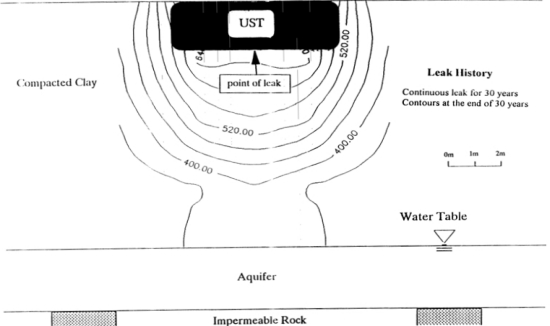
Gasoline contours in ppm after 30 years of leaking into a clayey soil [1].

The contours of total gasoline fraction and its components after 30 years of continuous leakage in a clay soil are shown in Figure 3 [1]. Note the gasoline has moved laterally to a considerable distance and also moved above the location of the leakage source (against gravity) which can only occur due to molecular diffusion. Since the clay soil is compacted wet of optimum (*S*_r_ = 60%) and the hydraulic conductivity of the clay was very low (*k*_w_ = 2 × 10^−7^ cm/sec), the physical process of advection played a minor role in the movement of contaminants through the clay. The greater spread in lateral directions may be due to the anisotropy of the clay layer resulting from lower tortuosity of the path in the lateral direction as the result of manual and centrifugal compaction of the clay. Even though the same volume of gasoline was stored in this tank as that in test with silty soil, nearly 1/3 of it did not leak. It indicates that the rate of leakage may be governed by both the soil type underneath a UST and the size of the hole. Lo *et al.* [24–26] confirmed the above observations.

### 2.3. Simulation of LUST under Flowing Groundwater

**Figure 4.**
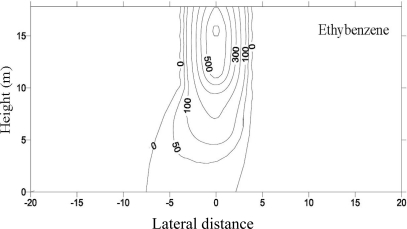
Centrifugal test results: spatial distribution of LNAPL concentration in unsaturated soil after 1-year migration (groundwater flow, unit: ppm) [28].

**Figure 5.**
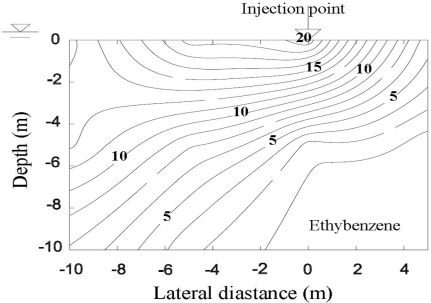
Centrifugal test results: spatial distribution of LNAPL concentration in water phase after 1 year migration (groundwater flow, unit: ppm) [28].

Figures 4 and 5 show the spatial distribution of LNAPLs in the unsaturated zone and water phase after one year migration with groundwater flowing [28]. Apparently, groundwater flow expedited the transport of LNAPL in subsurface system. Although the LNAPL spatial distribution in the upper part of the unsaturated zone was little affected by the groundwater flow, the contamination pattern near the capillary fringe is quite different from that under the stationary groundwater condition. In groundwater system, the hydrodynamic dispersion due to water flow greatly accelerated migration of dissolved LNAPL, and the contaminated area is much larger. Similar findings were also obtained from the numerical model simulation [28,29]. It can be extrapolated from the above tests that LNAPLs will continue to move along the capillary fringe and more dissolved components will migrate into the groundwater.

Hence it can be concluded that for granular soils the movement was advective and the free product formed a pool on top of the water table directly below the UST. However for fine grained soils instead of the pooling of free products the components of gasoline spread laterally due to the transport in anisotropic soils. When remediating LUSTs in granular soils, first the pooled free product should be extracted by pumping followed application of remediation technologies listed below to remediate gasoline contamination. However, when remediating LUSTs in cohesive soils, remediation technologies listed below are used to remediate gasoline contamination. The flowing groundwater would exacerbate the problem and the extent of contamination can be simulated using numerical models with known ground water flow rates and directions.

## 3. *In-situ* Remediation of Gasoline

**Figure 6.**
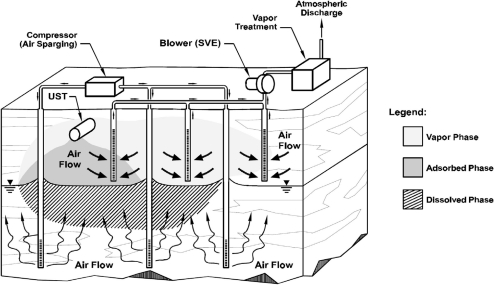
Schematic of coupled IAS and SVE remediation [4].

There are numerous cleanup technologies for gasoline-contaminated sites such as soil vapor extraction (SVE), *in-situ* air sparging (IAS), bio-sparging, land-farming, bio-piles, bio-venting, low-temperature thermal desorption, and natural attenuation [4]. Figure 6 shows schematic of coupled IAS and SVE remediation, two of the most frequently used technologies [4]. Laboratory tests were performed to investigate the efficiency of SVE and IAS and its influencing factors [30–38]. Hu *et al.* [39] simulate the air flow in fine sands under different sparging pressure. However, the experiments were conducted under 1 *g* and the *in-situ* stress condition could not be simulated. Centrifugal technology provides a powerful tool to simulate air flow and contaminant transport in porous media.

### 3.1. SVE Remediation

The SVE technology involves drawing or injecting air through the soil vadose zone above the water table. It is an effective soil remediation method to remove volatile organic compounds (VOCs) mainly existing in LNAPLs. The remediation takes place by desorption and vaporization of VOCs in unsaturated soils and by carrying them in the gas-phase out of the contaminated soils.

**Figure 7.**
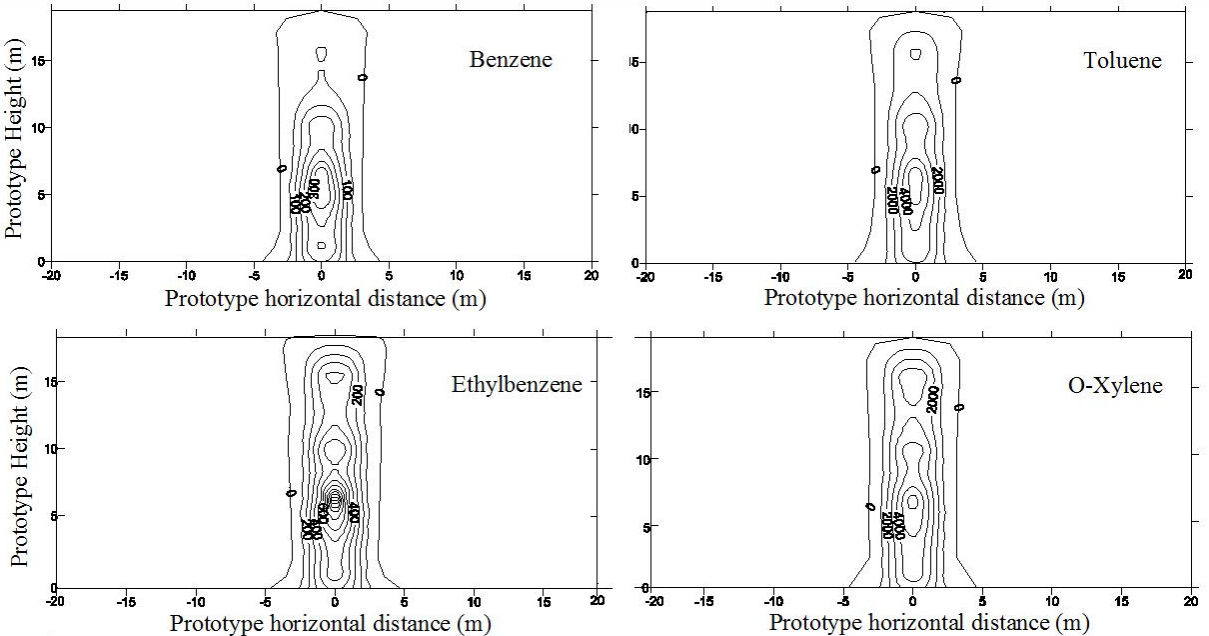
BTEX concentration after 1 year migration in fine sand (concentration in ppm) ([15], with permission from ASCE).

**Figure 8.**
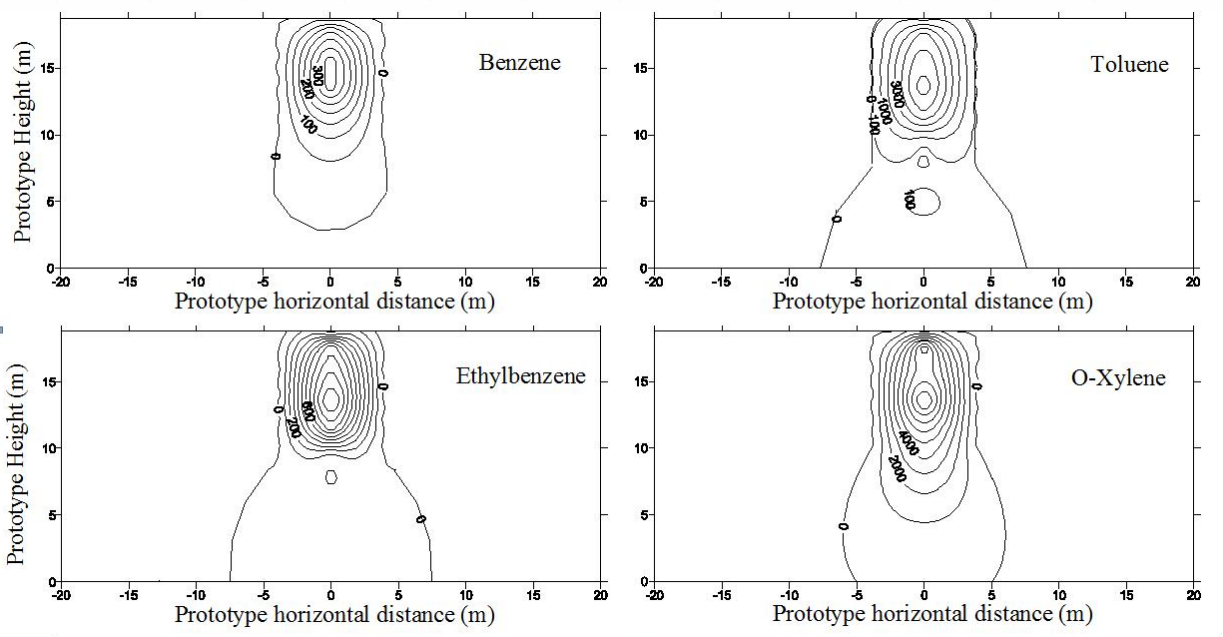
BTEX concentration after SVE for 2 months (concentration in ppm) ([15], with permission from ASCE).

**Figure 9.**
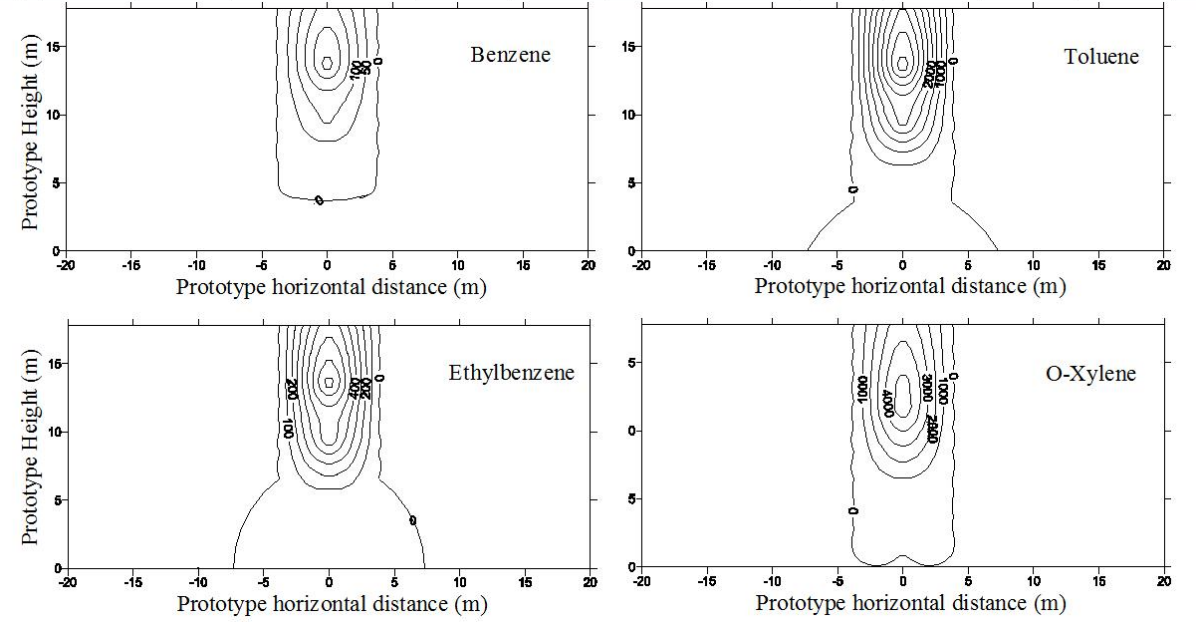
BTEX concentration after SVE for 4 months (concentration in ppm) ([15], with permission from ASCE).

Centrifuge tests were conducted to investigate the LNAPL migration behavior during SVE remediation process. During the first year of migration, as shown in Figure 7, LNAPL concentrated along the vertical line of the leaking point and lateral spreading up to a radius of 4.0 m around the line of the leaking point was detected [15]. For the SVE, the compressed air was injected into the soil mass, and air flow was intrigued from the injection pipe with higher pressure. The contaminants, specifically the BTEX from the contaminated soil were extracted from the air phase. The spatial distributions of the BTEX components before SVE and after SVE for 2 months and 4 months are presented in Figures 8 and 9 [15]. It is quite clear that the high concentration zone moved upwards from 5–6 m above the water table before remediation to about 13 m above the water table after SVE remediation. This implies that most of contaminants moved upwards to the ground surface with the air flow in soil mass. The highest concentration values changed slightly. However the extent of contamination decreased significantly, and the severely contaminated soil mass near the water table was remediated by the SVE process.

The residual amount of BTEX after SVE remediation for 2 and 4 months is also listed in Table 1 [15]. Approximately 47% of the BTEX mixture was removed from the contaminated site after 2 months of SVE. Only 13% of benzene was left in soil mass, while the residual percentages were 52%, 64%, and 58% for toluene, ethylbenzene, and *o*-xylene, respectively. This can be due to the fact that the vapor pressure of benzene is the highest among BTEX components, so benzene is much easier to be extracted into air phase. When SVE continued for 4 months, 68% of the total contaminant was removed. With additional clean air injected into the soil mass for flushing, more volatile organic contaminants can be further removed. Moreover, the vapor pressure of contaminant components is found to play an important role on the effectiveness of SVE. It appears that substantial amount of BTEX in soil phase and free phase was extracted into air phase and escaped from soil mass. These results demonstrated that SVE is an effective soil remediation technology for volatile contaminants.

### 3.2. IAS Remediation

*In-situ* air sparging (IAS) is an *in-situ* soil/groundwater remediation technology, which involves the injection of air under pressure into a well installed into the saturated zone. Air sparging technology extends the applicability of soil vapor extraction to saturated soils and groundwater through physical removal of volatilized groundwater contaminants and enhanced biodegradation in saturated and unsaturated zones. Air/oxygen injected below the water table volatilizes contaminants that are dissolved in groundwater, existing as a separate aqueous phase, and/or sorbed onto saturated soil particles. The volatilized contaminants migrate upward into the vadose zone, where they are removed, and generally using soil vapor extraction techniques. In addition to this air stripping process, air sparging also promotes bio-degradation by increasing oxygen concentrations in the subsurface, stimulating aerobic biodegradation in the saturated and unsaturated zones. The success of air sparging as a remedial technology for treatment of contaminated aquifers is well documented. However, there is no consensus, to date, on the mechanisms that control the flow of injected air through the saturated ground and the subsequent removal of contaminants. Therefore, it is difficult to develop design guide for site implementation. Currently, only qualitative results from laboratory experiments are available to predict the zone of influence of a sparging well. Air sparging systems must be designed with adequate air flow rates and pressures for the effective removal of contaminants from the site.

**Figure 10.**
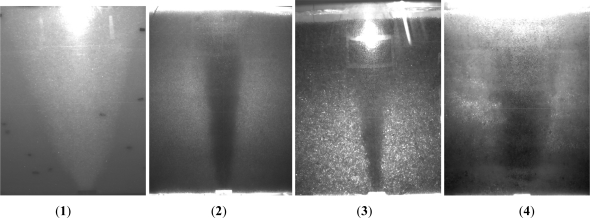
Zone of influence in centrifuge tests at 15 *g* (**1**) 0.8–1.0 mm (**2**) 1.5–2.0 mm (**3**) 4.0–5.0 mm (**4**) 0.8–5.0 mm.

**Figure 11.**
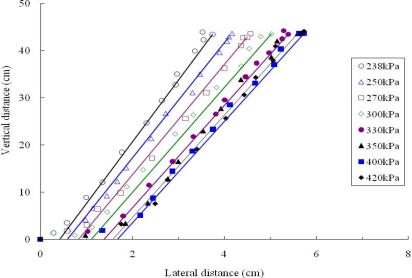
Zone of influence with different sparging pressures in 1.5–2.0 mm sample under 50 *g*-level tests.

Based on the centrifuge tests employing glass beads as soil, Hu *et al.* [40,41] simulated the air sparging under a wide range of sparging pressures and centrifuge *g* levels. Four samples with different particle size distribution were prepared and tested under different *g*-levels of 15, 30, 40 and 50. The physical properties of glass beads used for centrifuge tests are shown in Table 2. The ZOI is cone-shaped for different samples under various *g*-levels. Figure 10 shows the photos of stable ZOI in centrifuge tests at 15 *g*. It can be shown that for the uniform samples (Samples 1–3), the ZOI decrease with the increase of particle size. Figure 11 shows the boundary of ZOI under different air pressure for 1.5–2.0 mm sample under different air pressure in centrifuge tests at 50 *g*. The ZOI expanded with the increase of the inject air pressure at early stage. While the sparging pressure is higher than the critical value, the ZOI decreased a little and then remained constant. The critical value of the air pressure is termed “critical sparging pressure”. Table 3 gives the value for critical sparging pressure for different samples at different *g*-level. It is show that the particle size has little influence on the critical sparging pressure. The *g*-level has substantial influence in critical sparging pressure, and the difference of critical sparging pressure and pore-water pressure increases with the increase of *g*-level. The stable ZOI can be described by use of the lateral intrusion around the air injection point and the cone angle between the vertical axis and the boundary of ZOI. The volume of ZOI decreased with the increase of soil particle size and g level.

Based on the above discussion, it can be concluded that though IAS is a well developed technology, there are no design guides and hence the remediation is case specific. There are additional centrifugal tests being conducted with theoretical model development to rectify this situation [42,43].

## 4. Remediation and Reuse of Gasoline Contaminated Soils

To permanently close an UST, owners and operators must empty and clean it by removing all liquids and accumulated sludge. All tanks taken out of service permanently must also be either removed from the ground or filled with an inert solid material. In many instances UST owners found their tanks had leaked and the soil was already contaminated. Most of the states have developed allowable contamination levels for soils. Hence, the excavated contaminated soils due to leaking USTs and spills are required to be removed and isolated in a designated area for proper disposal. Though these soils are not classified as hazardous waste, they cannot be used as clean fill, as they are classified as solid waste.

For construction use of petroleum contaminated soils, soils should first be identified and classified. Ratnaweera and Meegoda [44] developed a methodology to identify and classify petroleum contaminated soils. Meegoda *et al.* [45] showed that petroleum contaminated soils can be used for construction of highway pavements. They studied the compaction characteristics of three soils: clay of high plasticity, clay of low plasticity and silty clay, containing 3%, 6%, and 12% motor oil. These mixtures were found to be effective and safe for using as a sub base material. The stabilization process produced physically, mechanically and chemically new soil mixtures.

Several other studies have attempted to use petroleum contaminated soils (PCSs) in hot mix asphalt concrete (HMA). Meegoda *et al.* [46–48] investigated the incorporation of PCSs into HMA in New Jersey, USA, and found that it was possible to include up to 35% PCSs in the mix. The mixes were evaluated for stability using the Marshall method and durability using the tensile strength ratio for conditioned and unconditioned specimens. Conditioning was done by wet-dry and freeze-thaw cycles. The stability results for PCSs compared with control mixes indicated a much better paving material, while the durability was found to be the same as the control mix. The leachability tests showed neither significant concentrations nor significant increase in concentrations with time. Meegoda *et al.* [46–48] also showed that the tensile strength ratio (TSR) for HMA mixes using PCSs were not significantly different from that for the control mix. They showed that the saturated hydraulic conductivity values of all HMA mixes with PCSs were less than 2 × 10^−6^ cm/s. To investigate the effect of using PCSs in HMA on air quality, a field study was performed where the air quality was monitoring by measuring the release of volatile organic compounds during HMA production. The results indicated that the concentration of target chemicals such as toluene, xylene and hexane in the air were less than 1 ppm and concentration of VOCs were lower than the allowable specification in New Jersey (<250 ppm).

In a field demonstration, Meegoda *et al.* [49] designed and constructed a base course of a two lane highway with heavy vehicular traffic with petroleum contaminated soils. Approximately 17,000 tons of HMA concrete was made with PCSs. Prior to the field demonstration a design mix containing PCS was obtained and evaluated for suitability by testing the Marshall stability, durability and permeability. The performance of the highway pavement was evaluated immediately after construction and one year later using a heavy weight deflectometer. The heavy weight deflectometer results showed that the mechanical performance of HMA with remediated PCSs was comparable to that without PCSs. Both full-scale field and laboratory tests proved that PCSs can be used in production and paving of HMA concrete.

Based on the above successful field and laboratory tests, Meegoda *et al.* [49] proposed that PCSs can be used in production and paving of HMA concrete. The above results enabled the New Jersey Department of Transportation (NJDOT) to modify their standard specification to include 20% PCSs in bituminous stabilized base course or use as a soil aggregate. However, the PCSs should comply with the following aggregate quality and gradation requirements of NJDOT:

**Table:** 

Mica..................................................	20.0% Maximum
Water Absorption..............................	2.0% Maximum
Sodium Sulfate Soundness, Loss......	5.0% Maximum
Plasticity Index..................................	Non-plastic
Clay and clay lumps..........................	5.0% Maximum

The cold mix asphalt concrete (CMA) is made by mixing asphalt emulsions with virgin aggregates. The end product, cold mix asphalt concrete, is usually spread, graded and compacted to form a strong asphalt concrete pavement. The mixing water in the asphalt emulsion is evaporated to form asphalt concrete. The cold mix asphalt concrete is used for paving base courses of asphalt concrete roads, parking lots and low volume roads. It is possible to incorporate PCSs into CMA but strength, durability and other engineering properties of asphalt concrete should be acceptable. These properties are needed for the effective use of cold mix asphalt concrete with petroleum contaminated soils as construction material. Meegoda [50] performed a research study to evaluate the design parameters for asphalt pavements, and mechanical properties of cold mix asphalt concrete with petroleum contaminated soils. In this research, several commercially available asphalt emulsions were used to make the CMA. The best emulsion type for six different petroleum contaminated soils was selected. Test results showed that the best emulsion type was cationic slow setting (CSS-1h). The Marshall stability tests indicated that the CMA with PCS was strong enough to be used in low volume roads (more than 1,000 lbs of Marshall stability). The wet/dry durability and freeze/thaw durability of the CMA with PCSs showed durability values that were comparable to those of a regular CMA without PCSs. The CMA with PCSs had hydraulic conductivity values comparable to those of asphalt concrete. There was no leaching of U.S. Environment Protection Agency priority pollutants from CMA mixes. Based on the test data, Meegoda [50] concluded that asphalt emulsions could stabilize and solidify PCSs to produce construction material. The unit cost values showed that it was also a cost-effective recycling process. Based on its simplicity, cost of production and end use as a construction material, the CMA technology for PCSs surpasses all other treatment, storage, and disposal methods for petroleum-contaminated soils [51]. If 5% petroleum contaminated soils is included in hot mix asphalt (HMA) concrete, such use can save the producer US$0.45 per ton of HMA as virgin aggregate costing US$9.00 per ton is replaced with petroleum contaminated soils. If one includes a US$100.00 per ton solid waste disposal cost, there is a US$5.00 saving per ton of asphalt produced.

## 5. Summary and Conclusions

Centrifugal model tests were performed to simulate the LNAPL transport behavior in unsaturated soil and groundwater. In granular soils gasoline moved due to the physical process of advection and was retained as a pool of free product above the water table. In clay soils, there was a spread of contaminants in the lateral direction. The spread of gasoline depended on the anisotropy of the soil. Also the contour profile of a given chemical within a clay soil depends on its water solubility, and its fraction in the gasoline. Amount leaked depends on both the type of soil underneath the USTs and the amount of corrosion. The flowing groundwater exacerbated the problem.

Centrifugal test results indicated that SVE was an effective method to remove LNAPLs from contaminated soils. After SVE for two months for the testing case, 47% of BTEX was removed from the unsaturated soil; after four months of SVE, nearly 68% of contaminant was extracted from the contaminated soil mass. The determination of the optimum air pressure, pipe spacing and location, and operation duration for the SVE technology need further investigation. The vapor pressure of each contaminant is a significant factor affecting the efficiency of SVE technology.

The knowledge of air flow pattern and extent of the zone affected by the injected air is not well understood in the designing IAS system for soil remediation. According to the centrifugal test results, the zone of influence (ZOI) during air sparging is in a truncated-cone shape under various *g*-levels, which can be expressed by the lateral intrusion around the air injection point and the cone angle between the vertical axis and the boundary of ZOI. Though IAS is a well developed technology, there are no design guides and hence the remediation is case specific. Additional centrifugal tests have been conducted with theoretical model development to rectify this situation.

Excavated PCSs from leaking UST can be used in highway industry as construction materials, specifically as sub-base material or added to make HMA and CMA concrete. Results from an in-depth laboratory and field studies showed that up to 35% of PCSs can be added based on the total weight of the aggregates to produce HMA as well as CMA concrete.

## Figures and Tables

**Table 1.** Mass balance of BTEX in centrifuge tests ([15], with permission from ASCE).

**Contaminants**	**Migration for 1 year**	**SVE for 2 months**	**SVE for 4 months**
**Benzene**	46%	13%	9%
**Toluene**	88%	53%	32%
**Ethylbenzene**	90%	64%	41%
***o*-Xylene**	100%	58%	36%
**Total BTEX**	88%	53%	32%

**Table 2.** Physical properties of glass beads used in tests.

**Sample No.**	**Particle size (mm)**	**Dry density (g/cm^3^)**	**Porosity (%)**
**1**	0.8–1.0	1.617	35
**2**	1.5–2.0	1.684	33
**3**	4.0–5.0	1.676	33
**4**	0.8–5.0	1.953	22

**Table 3.** Critical sparging pressure from centrifuge tests.

**Sample No.**	***g*level**	**Static water pressure (kPa) (1)**	**Critical AS pressure (kPa) (2)**	**(2)-(1) (kPa) (3)**
**1**	15	64.8	100	35.2
	30	112.4	170	57.6

**2**	15	68.1	105.0	36.9
	30	134.2	180.0	45.8
	40	170.7	240.0	69.3
	50	204.5	300.0	95.5

**3**	15	72	110.0	38.0
	30	137.5	180.0	42.5
	40	178.3	230.0	51.7
	50	211.6	290.0	78.4

**4**	15	66.9	100.0	33.1
	30	129.8	180.0	50.2
	40	168.3	240.0	71.7
	50	203.3	300.0	96.7
